# Biomimetic Electrodynamic Metal‐Organic Framework Nanosponges for Augmented Treatment of Biofilm Infections

**DOI:** 10.1002/advs.202408442

**Published:** 2024-10-18

**Authors:** Yanmin Wang, Wei Guo, Kai Zhang, Zhiwen Liu, Xiaoguang Dai, Zhuangzhuang Qiao, Xiaokang Ding, Nana Zhao, Fu‐Jian Xu

**Affiliations:** ^1^ Key Laboratory of Biomedical Materials of Natural Macromolecules (Beijing University of Chemical Technology Ministry of Education) Beijing Laboratory of Biomedical Materials Beijing University of Chemical Technology Beijing 100029 China; ^2^ College of Materials Science and Engineering Beijing University of Chemical Technology Beijing 100029 China; ^3^ Quzhou Institute for Innovation in Resource Chemical Engineering Quzhou 324000 China

**Keywords:** electrodynamic therapy, extracellular vesicles, metal–organic framework, neutralizing toxins

## Abstract

Electrodynamic therapy (EDT) is a promising alternative approach for antibacterial therapy, as reactive oxygen species (ROS) are produced efficiently in response to an electric field without relying on endogenous H_2_O_2_ and O_2_. However, the inherent toxicity of metallic catalysts and numerous bacterial toxins during the therapeutic process still hinder its development. Herein, biomimetic metal–organic (MOF@EV) nanosponges composed of ginger‐derived extracellular vesicles (EVs), and electrodynamic metal–organic frameworks (MOFs) are developed for the eradication of bacterial infections and the absorption of toxins. The prolonged circulation time of MOF@EV in vivo facilitates their accumulation at infection sites. More interestingly, MOF@EV can behave as nanosponges and effectively prevent host cells from binding to bacterial toxins, thereby reducing damage to cells. Subsequently, the MOF@EV nanosponges are discovered to work as electro‐sensitizers, which is confirmed through both theoretical calculation and experimental verification. As a result, ROS is continuously produced under the electric field to achieve effective EDT‐mediated bacterial eradication. Meanwhile, the treatment process of MOF@EV in vivo is visualized in mice infected with luciferase‐expressing *Staphylococcus aureus* (*S. aureus*), and excellent biofilm eradication capacity and detoxification efficiency are demonstrated in a subcutaneous abscess model. This work provides a promising strategy for the treatment of bacterial infections.

## Graphical abstract

The inherent toxicity of metallic catalysts and numerous bacterial toxins during the therapeutic process still hinder the development of Electrodynamic therapy. Herein, biomimetic metal–organic (MOF@EV) nanosponges composed of ginger‐derived extracellular vesicles and electrodynamic metal–organic frameworks are developed for the eradication of bacterial infections and the absorption of toxins.

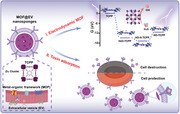

## 1 Introduction

Bacterial infections remain a significant global health concern, and the emergence of antibiotic‐resistant bacteria has led to an urgent need for novel non‐antibiotic treatment strategies.^[^ ^1^ ^]^ Tremendous observations have manifested that reactive oxygen species (ROS) – mediated therapeutic methods demonstrate potential in antibacterial therapy, as ROS are highly reactive molecules that are able to damage cell membranes, DNA, and proteins, ultimately leading to bacterial eradication, such as photodynamic therapy (PDT), sonodynamic therapy (SDT), and electrodynamic therapy (EDT).^[^ ^2^ ^]^ Among them, EDT utilizes an electric field to activate electro‐sensitizers to convert H_2_O into ROS without the limitation of the endogenous hydrogen peroxide (H_2_O_2_) and oxygen (O_2_), offering unique advantages in combating bacterial infections.^[^ ^3^ ^]^ Currently, platinum (Pt)‐based noble metal nanoparticles are often employed as electro‐sensitizers for EDT‐mediated antibacterial therapy. Our group has constructed Pd‐Pt nanosheets as efficient electro‐sensitizers for the eradication of bacteria, which can facilitate the production of ROS through a high affinity for bacteria to promote H_2_O dissociation on their surfaces.^[^ ^4^ ^]^ However, the intrinsic toxicity of metal‐based electro‐sensitizers severely hampers EDT‐mediated antibacterial treatment in clinical applications. Therefore, the development of safe electro‐sensitizers is still a major challenge in achieving efficient EDT against bacterial infections.

Metal‐organic frameworks (MOFs) are porous nanomaterials composed of organic molecules and inorganic metal ions or clusters. MOFs have received considerable attention in the field of antibacterial infections due to the diversity of organic molecules and metal ions, and the infinite possible structures.^[^ ^5^ ^]^ Recently, porphyrin‐based MOFs have been widely employed as photosensitizers/sonosensitizers in antibacterial treatment due to their high capacity to generate ROS,^[^ ^6^ ^]^ which has never been utilized as nano‐electrosensitizers to combat bacterial infections to date. Notably, benefiting from the unique large conjugated π‐systems and semiconductor properties, porphyrins are widely used in electrocatalytic reactions, such as water decomposition, carbon dioxide reduction, and oxygen reduction.^[^ ^7^ ^]^ Thus, we can rationally hypothesize that porphyrins‐based MOFs can serve as nano‐electrosensitizers for EDT‐mediated treatment of bacterial infections. Moreover, the porous structure of MOFs facilitates the diffusion of ROS, which is expected to lead to efficient delivery of ROS to enhance antibacterial therapy in vivo.

Bacterial infections can not only cause direct harm to the host organism but also primarily generate irreversible damage to normal cells caused by the abundant toxins secreted from bacteria.^[^ ^8^ ^]^ Numerous studies have demonstrated that the effective inhibition of bacterial toxins can greatly reduce the severity of bacterial infections.^[^ ^9^ ^]^ However, current methods to neutralize toxins rely on the design of molecular structures tailored to each specific toxin, limiting the efficiency of antibacterial treatment due to the diversity of bacterial species and the complexity of toxins.^[^ ^10^ ^]^ Thus, it is desirable to develop new strategies to neutralize a broad spectrum of toxins based on their functions. Typically, bacterial toxins exhibit high specificity and affinity during the recognition process to their target host cells, which is primarily attributed to their intimate interactions with specific receptors (lipids and proteins) on the cell membrane.^[^ ^11^ ^]^ For example, phosphatidylcholine as an important component of the cell membrane has been identified as the specific receptor of α‐hemolysin secreted from Gram‐positive *Staphylococcus aureus* (*S. aureus*).^[^ ^12^ ^]^ Plant‐derived exosomes possess the typical phospholipid membrane structure and preserve biological components,^[^ ^13^ ^]^ which are considered promising candidates for neutralizing various toxins while preserving the normal host microbiome. Thus, camouflaging nano‐electrosensitizers with ginger‐derived extracellular vesicles (EVs) can be an effective strategy to neutralize toxins, ultimately enhancing the antibacterial capabilities in vivo.

**Scheme 1:**
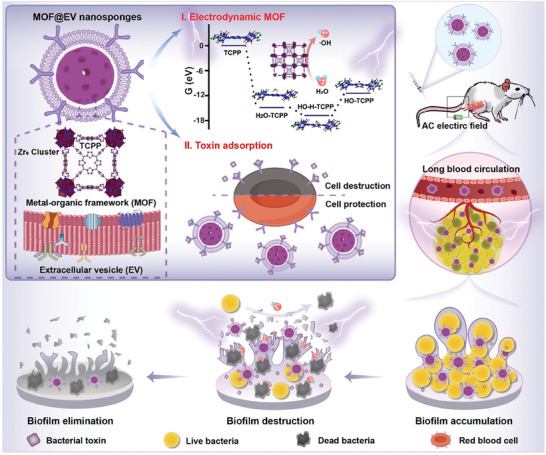
Schematic Illustration of the preparation of MOF@EV and its antibacterial mechanism.

Herein, MOFs with electrocatalytic activity were synthetized by Zr^2+^ and meso‐tetra (4‐carboxyphenyl) porphine (TCPP) through a simple solvent thermal method,^[^ ^14^ ^]^ followed by the coating of ginger‐derived EVs to fabricate the biomimetic multifunctional antibacterial nanosponges (MOF@EV) (**Scheme** **1**). The biomimetic nanosponges possess excellent toxin‐absorption capability and are capable of efficiently generating ROS under the action of an electric field. An appropriate negative potential of MOF@EV can prevent non‐specific protein binding and prolong its circulation time in vivo, thereby achieving effective accumulation at the bacterial infection sites. In addition, the results in vitro demonstrate that the MOF@EV can achieve effective antibacterial effects and reduce the hemolytic activity of bacterial toxins. Therefore, these biomimetic MOF nanosponges provide a promising nano‐electro‐sensitizer for EDT‐mediated antibacterial applications.

## 2 Results and Discussion

### 2.1 Preparation and Characterization of MOF@EV Nanosponges

**Figure 1:**
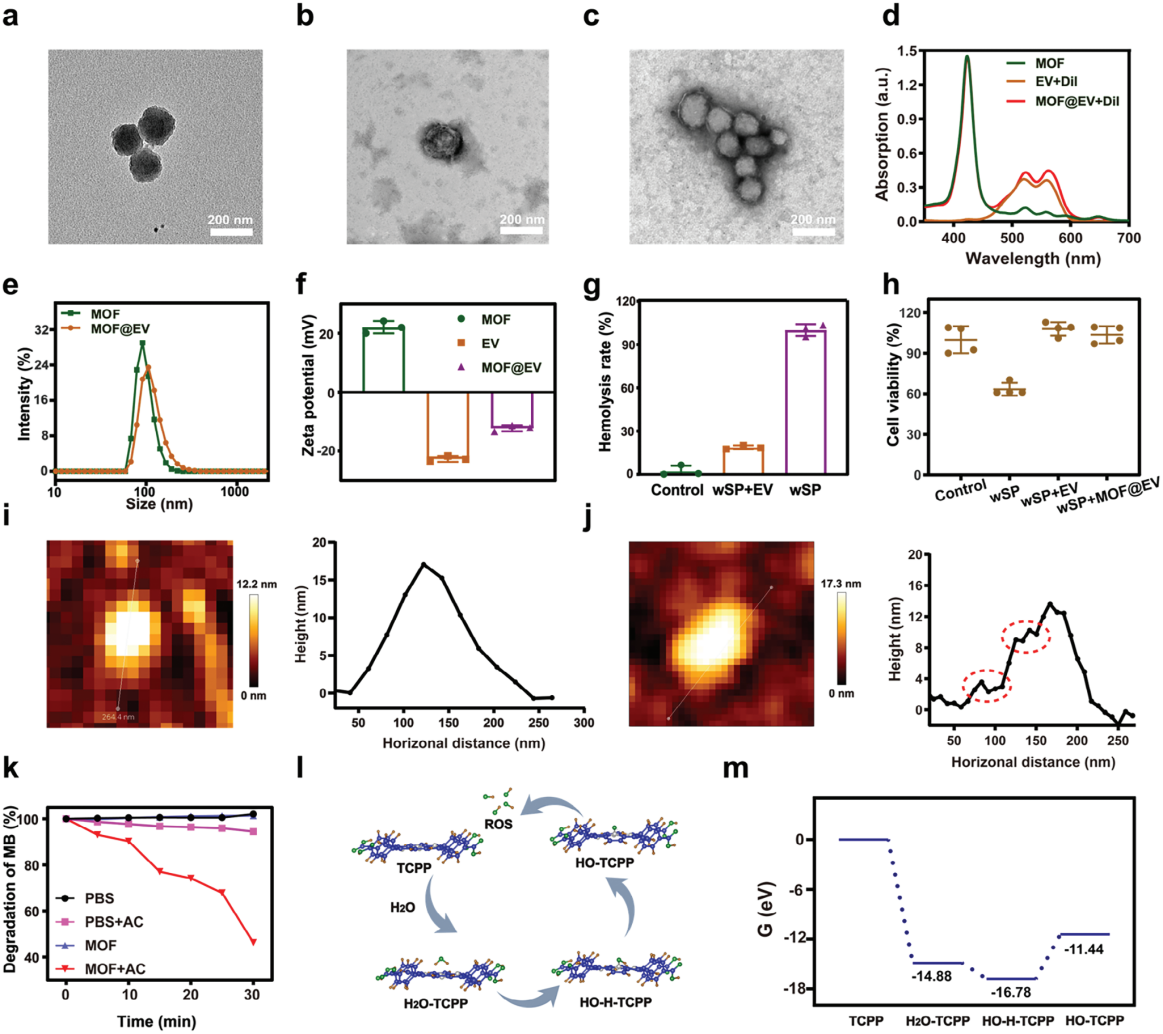
Transmission electron microscopy (TEM) images of a) MOF, b) ginger‐derived EVs, and c) MOF@EV. d) UV–vis absorption spectra of MOF, EV + Dil, and MOF@EV + Dil. e) Dynamic light scattering (DLS) analysis of MOF and MOF@EV. f) Zeta potential of MOF, ginger‐derived EVs, and MOF@EV (*n* = 3). g) Hemolysis ratio after different treatments (*n* = 3). h) Viability of L929 cells treated with the whole secreted proteins (wSP), EV, and MOF@EV (*n* = 4). Topology of EVs visualized by AFM i) before and j) after the treatment of toxins secreted from *S. aureus*. k) Degradation behaviors of MB under different treatments. l) Analysis of catalysis mechanism. The blue, gray, green, and brown balls represent C, N, O, and H atoms, respectively. m) The calculation of Gibbs reaction energy for ROS generation.

The preparation procedure of MOF@EV nanosponges (NPs) is illustrated in Scheme 1. As shown in **Figure** **1**a,b, transmission electron microscopy (TEM) images demonstrate that the synthesized MOF NPs exhibit a uniform spherical structure with an average diameter of ≈90 nm, while the EVs extracted from ginger present an obvious saucer‐like shape. The TEM image of MOF@EV clearly shows that the MOF was enveloped by the shell of ginger‐derived EVs (Figure 1c). The UV–vis absorption spectra of Dil‐stained EVs (EV – Dil) showed that resultant MOF@EV exhibited characteristic peaks of both MOF and stained EVs, indicating the successful construction of MOF@EV (Figure 1d). The size distribution of EVs extracted from ginger was characterized by Nanoparticle Tracking Analysis (NTA) (Figure S1, Supporting Information). In addition, the camouflage of EVs endowed the MOF@EV NPs with an increase in hydrodynamic diameter from ≈123.1 nm of MOF to ≈169.9 nm (Figure 1e). The zeta potentials of MOF and EVs were +22.02 and −22.70 mV, respectively. Notably, the zeta potential of MOF@EV NPs changed to −12.30 mV after coating EVs, which was more conducive to circulation in vivo compared with positive charges pure MOF NPs (Figure 1f). Moreover, the size of MOF@EV in PBS showed a negligible change after 5 days, demonstrating its structural stability (Figure S2, Supporting Information). Additionally, the result of sodium dodecyl sulfate‐polyacrylamide gel electrophoresis (SDS‐PAGE) analysis confirmed that the resultant MOF@EV retained the characteristic proteins of EVs (Figure S3, Supporting Information). These results demonstrated the successful construction of MOF@EV NPs.

### 2.2 Toxins Neutralization

The toxins released by *S. aureus* induce the formation of pores on the membrane to disrupt the integrity of cell membranes, thereby harming the host cells.^[^ ^15^ ^]^ Drawing the inspiration from the principles governing interactions between toxins and cell membranes, the capacity of MOF@EV to neutralize toxins secreted from *S. aureus* was investigated through hemolytic experiments and 3‐(4,5‐Dimethylthiazol‐2‐yl)‐2,5‐diphenyl tetrazolium bromide (MTT) analysis. As shown in Figure 1g, compared with the control group, the red blood cells incubated with the whole secreted proteins (wSP) from *S. aureus* exhibited a much higher hemolysis ratio, which was attributed to the damage of the bacterial toxins to cell membranes. Notably, when the red blood cells were co‐incubated with the wSP from the EVs pre‐treated *S. aureus*, a significant reduction in hemolysis ratio was observed (Figure S4, Supporting Information). Furthermore, the result of BCA protein assay demonstrated that the concentration of toxins secreted by bacteria significantly decreased after the co‐incubation with EVs compared with the control group (Figure S5, Supporting Information). Furthermore, MTT assay was employed to evaluate the degree of MOF@EV‐mediated alleviation of cytotoxicity in L929 cells induced by wSP from *S. aureus* (Figure 1h). The wSP extracted from *S. aureus* without any treatment showed obvious damage to L929 cells, exhibiting cell viability down to ≈64.42%. Excitingly, after pre‐treatment with EVs or MOF@EV, L929 cells co‐incubated with wSP demonstrated similar cell activity to the control group, further suggesting that the ginger‐derived EVs could neutralize toxins and thus attenuate the damage caused by wSP. Additionally, as shown in Figures 1i,j, pores on the surface of EVs after toxins treatment were characterized by atomic force microscopy (AFM). The result of height distribution implied that EVs were identified as spherical structures. In contrast, protrusion was detected in the presence of toxins. These results suggest that ginger‐derived EVs could efficiently neutralize bacterial toxins secreted from *S. aureus*, thus decreasing the damage to normal tissues and enhancing the bio‐safety of MOF camouflaged by EVs.

### 2.3 ROS Generation Performance of MOFs

Tremendous studies demonstrate that ROS with high cytotoxicity can destroy bacteria by decomposing essential biomolecules, including DNA, lipids, and proteins, leading to the death of bacterial cells.^[^ ^16^ ^]^ To investigate the electro‐driven catalytic activity of MOFs, methylene blue (MB) degradation experiments were conducted to characterize the ROS generation capability in a double salt bridge system under the square‐wave electric field (alternating current, AC). As shown in Figure 1k, the characteristic absorption peak of MB at 664 nm exhibited negligibly change receiving the single treatment of pure PBS, AC, or MOF, indicating that negligible ROS was generated under a single experimental condition of AC or MOF only. In contrast, an obvious degradation of MB could be observed in the presence of MOF and AC, which was attributed to the efficient ROS generation capacity of MOF under the treatment of AC. Furthermore, the depletion profile of MB displayed a time‐dependent behavior, indicating that extending the duration of electrical stimulation could enhance the ROS production efficiency of MOF. Afterward, density functional theory (DFT) calculations were used to explore the mechanism of ROS generation (Figure 1l,m).^[^ ^17^ ^]^ In the calculation model, porphyrin molecules were considered active sites, and H_2_O molecules can be dissociated triggered by AC. Initially, the adsorption energy of H_2_O on the pure MOF was −14.88 eV, which illustrated that H_2_O could absorb easily on MOF. Then, H_2_O started to disassociate and the energy of water molecule decomposition was −1.90 eV. Finally, the energy barrier of water decomposition absorbed on porphyrin was 5.34 eV. Meanwhile, the changes in fluorescence signal from terephthalic acid (TA) as a fluorescent probe to detect ·OH further confirmed the generation of ·OH when the MOF was treated with electrical stimulation (Figure S6, Supporting Information). These results demonstrated that MOFs could serve as nano‐electro‐sensitizers to effectively generate ROS for EDT‐mediated antibacterial treatment.

### 2.4 Antibacterial Performances of MOF@EV In Vitro

**Figure 2:**
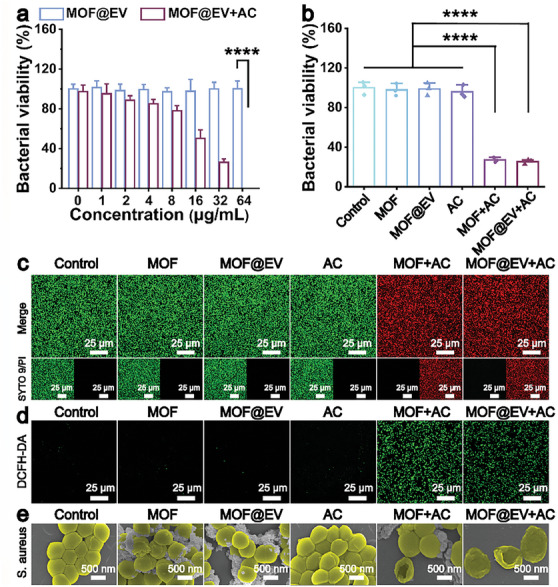
In vitro antibacterial activity of MOF@EV. a) Relative bacterial viability of *S. aureus* treated by MOF@EV at different concentrations with or without AC (*n* = 3). b) Bacterial viability (*n* = 3) and c) corresponding CLSM images of *S. aureus* receiving different treatments. d) ROS generation in *S. aureus* receiving various treatments by using a DCFH‐DA probe as an ROS tracker. e) SEM images of *S. aureus* after various treatments. Data are expressed as the mean ± SD (*n* = 3), ***p < 0.001, as determined by the one‐way ANOVA using the Tukey post‐test.

Encouraged by the effective ROS production capability of MOF@EV, the in vitro antibacterial performance of MOF@EV was further investigated via the plate counting method using *S. aureus* as a model.^[^ ^16^ ^]^ As shown in **Figure** **2**a; Figure S7 (Supporting Information), MOF@EV displayed negligible bactericidal activity even at a high concentration of 64 µgmL^−1^. However, in the presence of AC, the survival rate of *S. aureus* obviously decreased with the increase of MOF@EV concentration, ultimately reaching ≈99% at 64 µgmL^−1^. Compared with the control group, the growth of AC, MOF, and MOF@EV treatments showed no significant inhibitory effect on the growth of *S. aureus*. Notably, *S. aureus* incubated with MOF@EV and MOF exhibited similar bacterial counts under external AC treatment, suggesting the coating of EVs had a negligible effect on the MOF‐mediated EDT for antibacterial treatment (Figure 2b). Furthermore, a live/dead staining assay was carried out to visually observe the state of dead and living bacteria. The green fluorescence stained with SYTO 9 represented the living *S. aureus*, while the red fluorescence stained with propidium iodide (PI) represented the dead bacteria.^[^ ^18^ ^]^ As demonstrated in Figure 2c, the single treatment of PBS, MOF, MOF@EV, and AC had ignorable damage to *S. aureus*. In comparison, a large amount of red fluorescence signal could be observed in the *S. aureus* treated with MOF and MOF@EV in the presence of AC, which is consistent with the results of the plate counting method in Figure 2b. These results indicate the outstanding performance of MOF@EV in the treatment with AC in combating planktonic bacteria.

### 2.5 Electrodynamic Antibacterial Mechanism of MOF@EV

Benefiting from the rapid development of nanotechnology, researchers have focused on the in‐depth research on the electrical properties of nanomaterials and verified that oxidative stress is one of the essential mechanisms of nanoparticle‐mediated bactericidal behavior.^[^ ^19^ ^]^ To study the antibacterial mechanism of MOF@EV‐mediated EDT in vitro, 2′,7′‐dichlorofluorescein diacetate (DCFH‐DA), a common ROS tracker, was utilized to monitor the intracellular ROS production levels in *S. aureus*.^[^ ^20^ ^]^ As shown in Figure 2d, negligible green signals could be observed in *S. aureus* treated with only PBS, AC, MOF, or MOF@EV, suggesting that insignificant ROS was produced in these experimental conditions. In contrast, the co‐incubation of *S. aureus* and MOF induced a significant increase of green fluorescence intensity under the treatment of AC electric field, manifesting that MOFs could generate numerous ROS in the presence of AC electric field. Meanwhile, the expression level of ROS in the MOF@EV‐treated *S. aureus* under AC treatment was similar to that in MOF‐incubated *S. aureus* stimulated by AC, suggesting that EVs had no obvious impact on ROS production. These results demonstrate that the combination of MOF@EV and AC field could dramatically eradicate bacteria through ROS‐mediated treatment. Moreover, the integrity of membrane structures and morphological changes of *S. aureus* were observed by scanning electron microscope (SEM).^[^ ^21^ ^]^ As shown in Figure 2e, the integrated membrane and smooth surface of *S. aureus* were observed after receiving the mono treatment of PBS, MOF, MOF@EV, or AC electric field, implying insignificant damage to *S. aureus*. As expected, apparently shrinkage and deformation appeared on the membrane surface of *S. aureus* after treatment with MOF under AC electric field for 10 min, and a similar phenomenon occurred in the MOF@EV‐incubated *S. aureus* with the assistance of AC electric field. Taken together, MOF@EV‐mediated EDT achieved disruption of membrane structures through the generation of ROS to eradicate bacteria.

### 2.6 Antibiofilm Performance of MOF@EV

**Figure 3:**
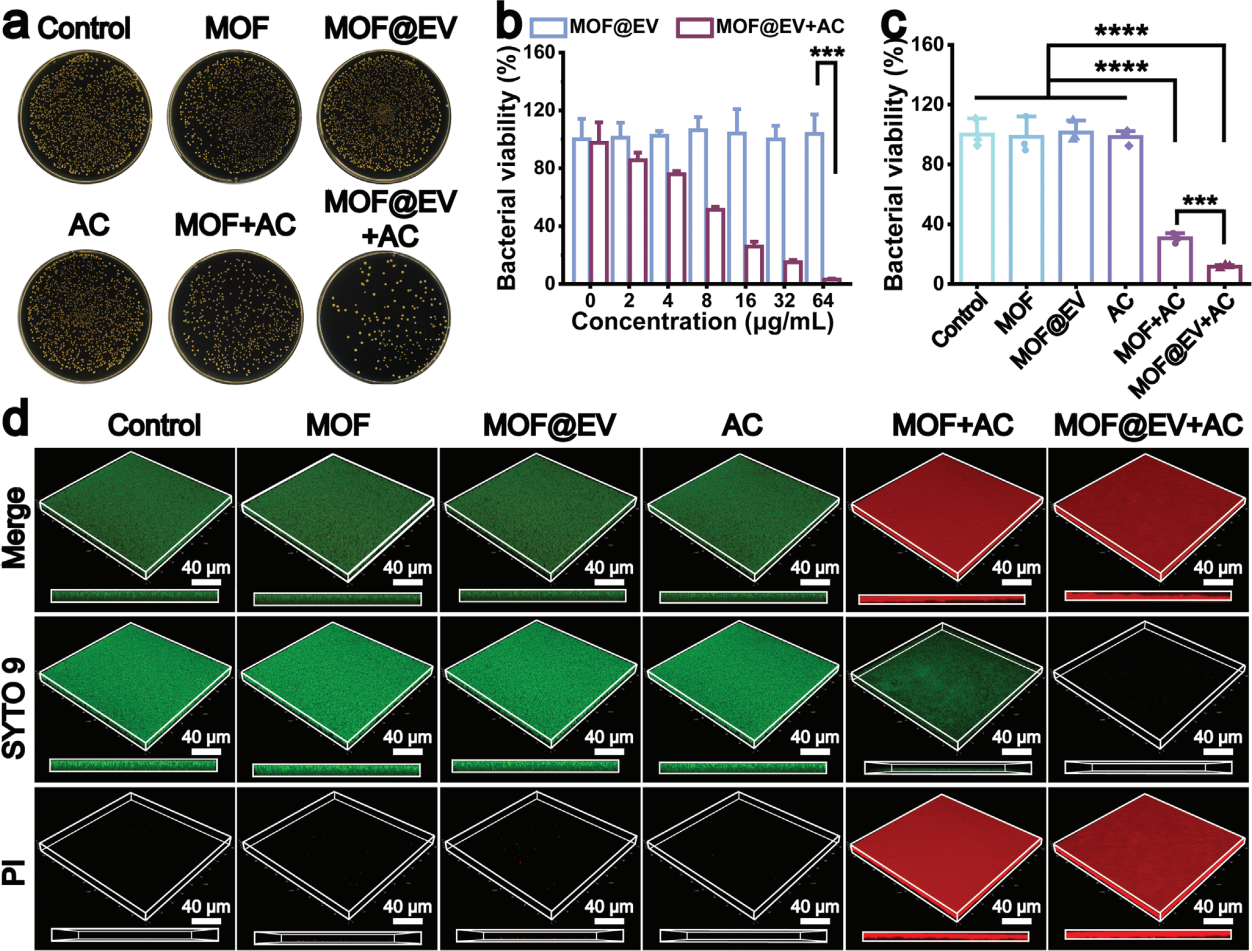
Antibiofilm performance of MOF@EV. a) The plates of bacteria detached from biofilms after receiving different treatments and c) the corresponding survival ratio of bacteria. b) Relative bacterial viability of *S. aureus* biofilms incubated with MOF@EV at different concentrations in the absence/presence of AC electric field. d) CLSM images of biofilms with different treatments through SYTO 9/PI fluorescence staining assay (*n* = 3). Data are expressed as the mean ± SD (*n* = 3), ***p < 0.001, as determined by the one‐way ANOVA using the Tukey post‐test.

Biofilm is often difficult to remove effectively and induces increased resistance to antibiotics, which has become a major health threat to bacterial infections.^[^ ^22^ ^]^ Herein, we utilized *S. aureus* biofilms as models to evaluate the antibiofilm ability of MOF@EV in vitro. The standard plate counting method was performed to verify the remaining amount of *S. aureus* in biofilms (**Figure** **3**a). With the assistance of an AC electric field, MOF@EV exhibited excellent anti‐biofilm capacity with the increase of MOF@EV concentration, and exceeding ≈99.9% of the bacteria were killed at MOF@EV content of 64 µgmL^−1^, whereas the MOF@EV had an insignificant effect on the elimination of biofilm in the absence of AC electric field (Figure 3b). Remarkably, the survival rate of *S. aureus* incubated with MOF@EV was ≈11.74%, which was substantially lower than that of MOF‐treated *S. aureus* (≈30.58%, Figure 3c). It's probably caused by the fact that the good biocompatibility of EVs promoted the penetration of MOF@EV in biofilm. Furthermore, live/dead double staining assays were employed to assess the antibiofilm efficiency of MOF@EV,^[^ ^23^ ^]^ and the 3D confocal laser scanning microscopy (3D CLSM) images of the *S. aureus* biofilms receiving various treatments were presented in Figure 3d. Nearly the entire green fluorescence associated with the living bacteria stained by SYTO 9 could be observed on the *S. aureus* biofilms treated with PBS, MOF, MOF@EV, or only under an AC electric field. Meanwhile, with the assistance of an external AC electric field, *S. aureus* biofilms co‐cultured with MOF and MOF@EV displayed plenty of red fluorescent spots assigned to PI‐stained dead bacteria, indicating the efficient antibiofilm efficacy of MOF@EV‐mediated EDT.

### 2.7 Antibiofilm Mechanism of MOF@EV

**Figure 4:**
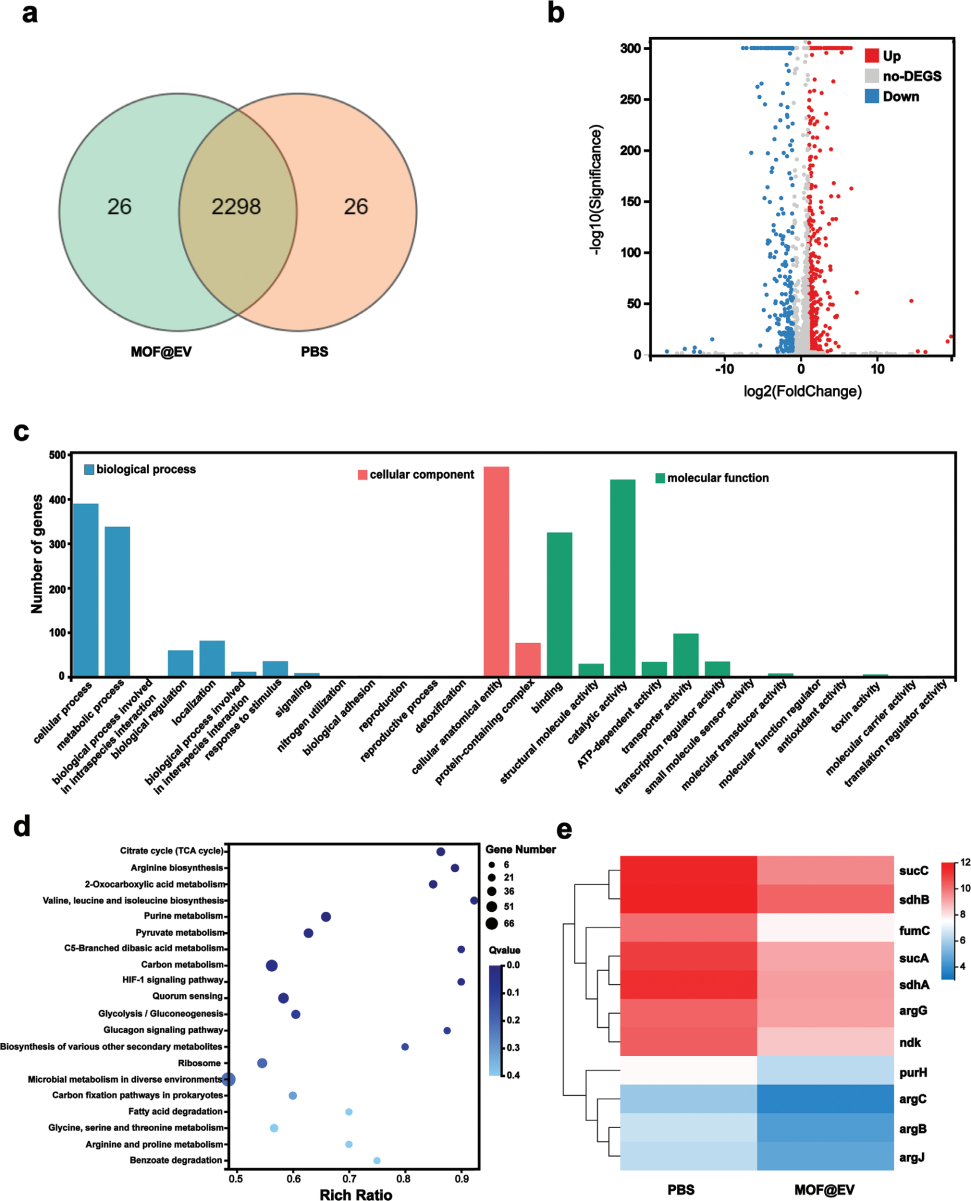
RNA‐seq analysis of biofilm after different treatments. a) Venn diagram of DEGs. b) Volcano plot of DEGs of *S. aureus* biofilms. c) GO annotation analysis and d) KEGG enrichment analysis of DEGs in biofilms. e) Heatmap of genes associated with biofilm‐related pathways.

To elaborate on the antibiofilm mechanism of MOF@EV, transcriptome sequencing (RNA‐seq) was conducted on *S. aureus* treated with MOF@EV and AC electric field. The gene expression profiles of biofilm cells after different treatments (PBS treatment as control) were analyzed. The differentially expressed genes (DEGs) between the two groups were presented in the Venn diagram (**Figure** **4**a). As depicted in the volcano plot (Figure 4b), 491 DEGs were upregulated and 277 DEGs were downregulated in the MOF@EV group in comparison with the control group. According to gene ontology (GO) analysis, DEGs between the control group and MOF@EV group were related to cellular components, biological processes, and molecular functions (Figure 4c). Additionally, the enrichment analysis result of Kyoto Encyclopedia of Genes and Genomes (KEGG) pathways exhibited that metabolism and virulence were affected (Figure 4d). The metabolism‐related pathways mainly included arginine biosynthesis, purine metabolism, glycine, serine, and threonine metabolism, which might help explain the antibiofilm mechanism. The regulation of energy metabolism‐related pathways such as citrate cycle (TCA cycle) broke energy metabolism and then inhibited biofilm growth. To further clarify antibiofilm properties based on EDT, hierarchical clustering heatmap analysis of biofilm‐related DEGs was performed (Figure 4e). The arginine biosynthesis was essential for bacterial growth.^[^ ^24^ ^]^ The result showed that the genes involved in arginine biosyntheses such as argB, argJ, argC, and argG were downregulated after the co‐incubation with MOF@EV, which might lead to the inhibition of arginine biosynthesis,^[^ ^25^ ^]^ further arginine depletion, and DNA is broken. Meanwhile, the expression of genes in the TCA cycle such as sucC, fumC, sucA, sdhA, and sdhB were downregulated, illustrating that the energy metabolism of biofilm was disrupted.^[^ ^26^ ^]^ The TCA cycle plays a vital role in providing energy for bacteria. After the energy metabolism was destroyed, the growth of *S. aureus* would be inhibited. Furthermore, the genes of purine metabolism such as purH and ndk were downregulated,^[^ ^27^ ^]^ and the expression of argJ associated with virulence was also downregulated. Briefly, the antibiofilm mechanism of MOF@EV might be attributed to inhibition of the TCA cycle, arginine biosynthesis, and purine metabolism.

### 2.8 Biocompatibility of MOF@EV

**Figure 5:**
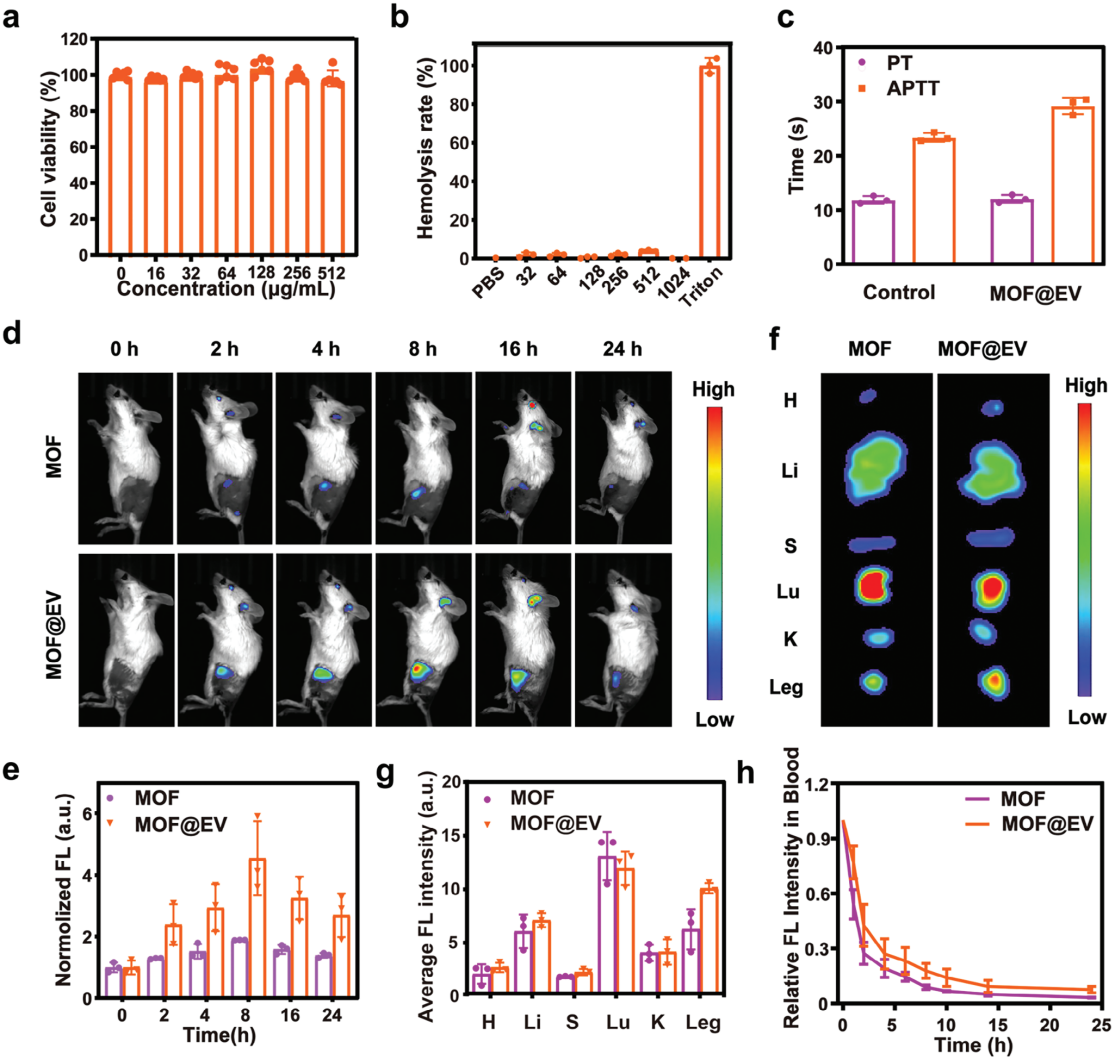
The biocompatibility of MOF@EV was evaluated in vitro and the biodistribution of MOF and MOF@EV NPs after tail vein injection in vivo. a) The cell viability of L929 cells treated with MOF@EV at various concentrations (*n* = 6). b) Hemolysis ratio of MOF@EV at different concentrations (*n* = 3). c) Blood coagulation of MOF@EV. Healthy mice acted as controls (*n* = 3). d) Fluorescence images of *S. aureus‐*infected mice at different time points after intravenous injection of MOF and MOF@EV, respectively. e) Corresponding fluorescence signal intensity in the infected region collected from mice after different treatments (*n* = 3). f) Fluorescence images (*n* = 3) and g) fluorescence intensity of major organs and infected tissues at 24 h post‐injection (*n* = 3). h) Fluorescence intensity of serum after injection of MOF and MOF@EV, respectively (*n* = 3).

The biocompatibility of MOF@EV was carefully evaluated using L929 cells incubated with MOF@EV at different concentrations prior to its further application in vivo. The cell viabilities were all above 90% even at the concentration of MOF@EV to 512 µg mL^−1^, indicating the negligible toxicity of MOF@EV (**Figure** **5**a). Meanwhile, the hemolytic activity of MOF@EV was studied by co‐culture of MOF@EV and red blood cells, and no significant hemolytic reaction was observed even at the MOF@EV concentration of 1024 µgmL^−1^, indicating the good hemocompatibility of MOF@EV (Figure 5b). Meanwhile, the results of prothrombin time (PT) and activated partial thromboplastin time (APTT) implied that no apparent differences could be observed between the control group and the MOF@EV group,^[^ ^28^ ^]^ revealing that MOF@EV had scarcely effect on the blood coagulation time (Figure 5c). These results revealed the biomimetic MOF@EV nanosponges have excellent in vitro antibacterial activity and good biocompatibility, showing promising prospects in in vivo antibacterial investigation.

### 2.9 In Vivo Accumulation of MOF@EV in the Infected Sites

The accumulation effect of MOF@EV is directly related to the eradication efficiency of NPs after intravenous administration. Using the *S. aureus‐*infected BALB/C mice as models, the fluorescence of porphyrin‐based MOFs was employed to monitor their biodistribution.^[^ ^29^ ^]^ As shown in Figures 5,e, weak fluorescence signals were observed at the infected sites of mice treated with MOF after injection, and subsequently, the fluorescence signal gradually disappeared, indicating the low accumulation efficiency of MOF in the infected sites. In contrast, after intravenous injection of MOF@EV, much stronger fluorescence signals were evidently observed and gradually increased over time in the bacteria‐infected tissues. The fluorescence signal at the infected tissue reached a maximum value at 8 h post‐injection and gradually decreased from 8 to 24 h. Subsequently, the infected tissues and primary organs were harvested after 24 h injection for ex vivo imaging to clarify the distribution profiles of MOF and MOF@EV. The fluorescence images and fluorescence intensity of major organs and infected tissues were presented in Figures 5f,g, indicating the efficient accumulation of MOF@EV in the *S. aureus‐*infected sites. Furthermore, the fluorescence intensity observed in the blood of the MOF@EV‐treated mice was much higher than that of MOF‐injected mice, suggesting that EVs could prolong the circulation time of MOF@EV in mice (Figure 5h).

### 2.10 In Vivo Electrodynamic Antibiofilm Performance of MOF@EV

**Figure 6:**
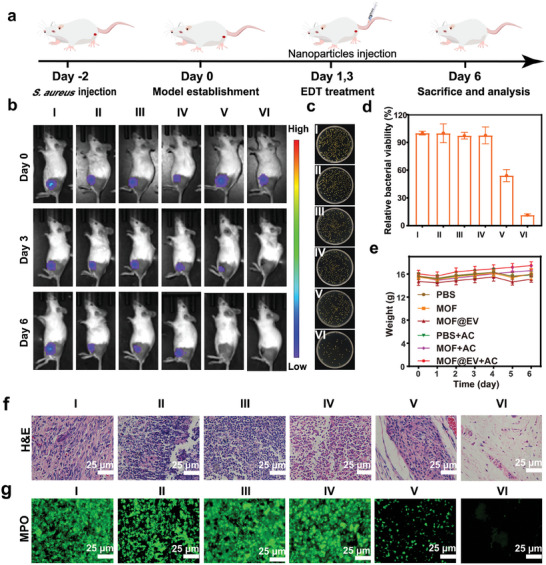
In vivo antibiofilm effect of MOF@EV using abscess model. a) Schematic diagram of a construction of the abscess model and the MOF@EV‐mediated treatment procedure. b) Photographs of mice after different treatments. c) Plates and d) survival rates of bacteria in the infected mice after different treatments (*n* = 3). e) Body weight changes of mice after receiving different treatments. Images of f) H&E and g) MPO staining images of skin tissues. The scale bar is 200 µm. (I) PBS group; (II) MOF group; (III) MOF@EV group; (IV) PBS + AC group; (V) MOF + AC; and (VI) MOF@EV + AC group.

Inspired by the excellent in vitro antibacterial capacity, good biocompatibility, and high accumulation efficiency of MOF@EV, a subcutaneous abscess model was established by subcutaneous injection of luciferase‐expressing *S. aureus* in the infected legs to investigate the intrinsic EDT activity of MOF@EV. The fluorescence signal associated with the luciferase‐expressing *S. aureus* was employed to monitor the therapeutic process in vivo (**Figure** **6**a). The antibacterial efficiency was obtained by the bioluminescence images (BLI) captured by the Xenogen IVIS spectrum imaging system, and the results are exhibited in Figure 6b. Apparently, the bioluminescence signal intensity of mice injected with PBS, MOF, MOF@EV, or AC alone showed a negligible reduction, demonstrating that they fail to combat bacterial infections. On the contrary, the bioluminescent signals obviously decreased in the bacteria‐infected site receiving the treatment of MOF + AC on the third day, verifying the antibacterial effects of MOF‐mediated EDT. Notably, a negligible bioluminescent signal was observed in the mice under the combination of MOF@EV and AC, indicating the better antibacterial effect of MOF@EV than MOF under AC exposure due to the improved accumulation efficiency and long circulation time of MOF@EV. Furthermore, the survival bacteria numbers in the tissues were validated by the plate counting method after 6 days of treatments to assess the sterilizing effect (Figures 6c,d). The lowest number of *S. aureus* was observed from the infected site under the combined treatment of MOF@EV and AC, which was consistent with the results of BLI. Additionally, the results of hematoxylin and eosin (H&E) staining presented in Figure 6f revealed that fewer inflammatory cells were observed in the infected tissues from the MOF@EV + AC group. In contrast, definite infiltration of neutrophils significantly appeared in the other groups, especially in the control group. Meanwhile, the images of immunofluorescence staining showed a similar trend and phenomena. As shown in Figure 6g, the myeloperoxidase (MPO) as a credible biomarker of neutrophil activation was utilized to characterize the level of inflammation in different groups, further demonstrating the superiority of the antibacterial effect of MOF@EV + AC. Remarkably, the expression levels of TNF‐α and IL‐6 in infected legs were significantly lower than the control group (Figure S8, Supporting Information), indicating that the degree of bacterial infection and inflammation decreased after MOF@EV + AC treatment.^[^ ^30^ ^]^ The potential toxicity in vivo of MOF@EV was evaluated using blood biochemical indexes. Compared with the normal group, the blood biochemical indexes of mice showed negligible changes after receiving the treatment of MOF@EV + AC, including white blood cell (WBC), lymphocyte (Ly), monocyte (MO), neutrophil (NEUT), red blood cell (RBC), hemoglobin (Hb), mean corpuscular volume (MCV), mean platelet volume (MPV), mean corpuscular hemoglobin (MCH), and red cell distribution width (RDW) (Figure S9, Supporting Information). Also, the alanine aminotransferase (ALT), serum aspartate aminotransferase (AST), creatinine (CREA), and urea (UREA) levels in the MOF@EV + AC group were equivalent to the control group (Figure S10, Supporting Information). Meanwhile, an insignificant change in body weight could be observed during the treatment procedure (Figure 6e), demonstrating that the MOF@EV had almost no side effects in vivo. H&E staining images of major organs also revealed that MOF@EV‐mediated EDT had no damage to organs (Figure S11, Supporting Information). These results suggest the biological safety of MOF@EV in vivo, which exhibited prominent potential for bacterial infection treatment.

## 3 Conclusion

In summary, biomimetic MOF@EV nanosponges were developed to absorb toxins and eliminate bacteria for anti‐infective therapy. MOF with the electrodynamic property was able to effectively generate ROS under the treatment of AC, demonstrating great potential in EDT‐mediated treatment of bacterial infections. Benefiting from the camouflage and protection of ginger‐derived EVs, the blood circulation time and biocompatibility of MOF@EV nanosponges were prolonged and improved, resulting in more accumulation at the infected sites. More importantly, EVs could neutralize toxins and reduce the damage of toxins to normal cells. With the assistance of AC, MOF@EV could continuously produce large amounts of ROS regardless of the microenvironment, which disrupted the structure of the membrane and resulted in bacteria elimination and biofilm eradication. The result of in vivo antibacterial experiments demonstrated that MOF@EV nanosponges‐mediated EDT could efficiently kill the luciferase‐expressing *S. aureus* and heal subcutaneous abscesses due to the excellent biocompatibility and bactericidal ability of MOF@EV. The currently constructed nanosponges presented a promising strategy for the eradication of bacterial infections.

## Conflict of Interest

The authors declare no conflict of interest.

## Supporting information



Supporting Information

## Data Availability

The data that support the findings of this study are available from the corresponding author upon reasonable request.
