# Supplementary material for: Biomimetic Electrodynamic Metal‐Organic Framework Nanosponges for Augmented Treatment of Biofilm Infections
Source: Adv Sci (Weinh). 2024 Oct 18;11(46):2408442. doi: 10.1002/advs.202408442 (PMC11633466; doi:10.1002/advs.202408442)
Supplement: Supplementary file 1 — Supporting Information [file ADVS-11-2408442-s001.docx]

Supporting Information

Biomimetic Electrodynamic Metal-Organic Framework Nanosponges for Augmented Treatment of Biofilm Infections

Yanmin Wang, ^a, b#^ Wei Guo, ^a, b#^ Kai Zhang, ^a, b, c*^ Zhiwen Liu, ^a, b^ Xiaoguang Dai, ^a, b^ Zhuangzhuang Qiao, ^a, b^ Xiaokang Ding, ^a, b^ Nana Zhao, ^a, b*^Fu-Jian Xu ^a, b*^

*^a^* Key Laboratory of Biomedical Materials of Natural Macromolecules (Beijing University of Chemical Technology, Ministry of Education), Beijing Laboratory of Biomedical Materials, Beijing University of Chemical Technology, Beijing, 100029, China

*^b^* College of Materials Science and Engineering, Beijing University of Chemical Technology, Beijing, 100029, China.

*^c^* Quzhou Institute for Innovation in Resource Chemical Engineering, Quzhou 324000, China

*^#^* These authors contributed equally to this work.

*To whom correspondence should be addressed

Email: zhangk@mail.buct.edu.cn (K. Zhang); [zhaonn@mail.buct.edu.cn](mailto:zhaonn@mail.buct.edu.cn) (N. Zhao); xufj@mail.buct.edu.cn (F. J. Xu)

**1. Experimental Section**

**1.1 Materials**

Benzoic acid (BA) was obtained from Beijing Yancheng Technology Co. Ltd. (Beijing, China). Zirconyl chloride octahydrate (ZrOCl_2_·8H_2_O) and Tetrakis (4-carboxyphenyl) porphyrin (TCPP) were purchased from Sigma-Aldrich Chemical Co. (St. Louis, MO). Methylene blue (MB) was provided by Energy Chemical (Shanghai, Chian). N, N-dimethylformamide (DMF) was purchased from Tianjin Fuchen Chemical Reagent Factory (Tianjin, China). 3-(4,5Dimethylthiazol-2yl) -2,5-diphenyl tetrazolium bromide (MTT) was brought from Sigma-Aldrich Chemical Co. (St. Louis, MO, USA). The strains of *S. aureus* (CMCC (B) 26003) were obtained from Promega (Madison, USA). The Live/Dead BacLight bacterial viability kit (L7012) was purchased from Invitrogen (Life Technologies, USA). 4’,6-diamidino-2-phenylindole (DAPI) was purchased from Beijing Solarbio Science & Technology Co., Ltd. Lysogeny broth (LB) medium for bacterial cultures was pre-treated by autoclaving at 120 ℃ for 20 min before use. Milli-Q Ultrapure water with a resistivity of 18.2 MΩ-cm was utilized in all experiments.

**1.2 Methods**

The morphology of MOF@EV was analyzed using Transmission Electron Microscopy (TEM) with a FEI Tecnai G2 analytical electron microscope operating at 200 kV (FEI Company, Hillsboro, OR). Scanning electron microscope (SEM) images were obtained by employing field emission SEM (Zeiss Supra 55) after platinum depositing for 120 s. UV-vis spectra was examined by SHIMADZU U3600 spectrometer (SHIMADZU, Japan). The size distribution and ζ-potential were measured using a Zetasizer Nano ZS instrument (Malvern Instruments, Southborough, MA). Confocal laser scanning microscope (CLSM) images were conducted on an oil immersed 63 × objective lens (Leica, SP8). EVs were centrifuged by ultracentrifugation (Beckman L-100XP, US).

**1.3 Synthesis of metal-organic frameworks (MOFs)**

PCN-224 was synthesized according to the previous literature.^[1]^ 10 mg of TCPP, 30 mg of ZrOCl_2_·8H_2_O, and 220 mg of BA were dissolved into 10 mL of N, N-dimethylformamide (DMF). The mixture was heated to 90 ℃ and stirred for 5 h. The PCN-224 nanoparticles were collected by centrifugation at 12,000 rpm for 15 min and washed with DMF and water. Finally, the product was suspended in water for further characterization and analysis.

- 1. **Preparation of ginger extracellular vesicles (EVs)**

Fresh ginger was purchased from a local supermarket and washed with phosphate-buffered saline (PBS). The ginger was then squeezed using a juicer, and the ginger juice was passed through gauze to remove any coarse residues. The mixture was centrifuged at 200 g for 10 min, 2000 g for 20 min, and 10,000 g for 30 min. Subsequently, the solution was subjected to ultracentrifugation at 150,000 g for 100 min to collect the precipitate.^[2]^ Then, the obtained precipitate was dissolved in PBS, and received sucrose gradient centrifugation to purify the ginger-derived EVs. The EVs were washed by ultracentrifugation in PBS to remove any remaining sucrose. Finally, the EVs were resuspended in PBS for further analysis. All procedures were performed at 4 ℃.

- 1. **Synthesis of MOF@EV**

The EVs were mixed with MOFs and sonicated through an Ultrasonic Dismembrator (LC-1000, Ningbo) under the special conditions (20 % amplitude, cycles of 3 s on/off for 10 min between each cycle at 4 ℃) to obtain MOF@EV. Finally, the resultant MOF@EV were purified by centrifugation at 12,000 g for 10 min to remove the unbound fragments, and then dispersed in PBS for further characterization and analysis.

**1.6 Measurement of ROS generation**

MB was employed as molecular probes to evaluate the ability of MOF@EV to generate reactive oxygen species (ROS), and the square-wave electric field (AC, 10 V, 10 mHz) were performed for various durations (0, 5, 10, 15, 20, 25, and 30 min) in the dark. Specifically, MOF@EV (200 μg/mL) was added to MB (10 mM, 20 μL), and the mixed solution was treated with the AC. The absorbance intensity of MB was monitored by UV-vis spectrophotometer at designated times. Additionally, pure MB, MB-AC, and MOF-MB served as control groups were also tested and evaluated using the same method.

**1.7 Antibacterial tests *in vitro***

*S. aureus* was selected as the model bacterium to assess the antibacterial efficiency of MOF@EV by using the spread plate method. *S. aureus* (1 × 10^8^ CFU/mL) was diluted to 1 × 10^5^ CFU/mL and blended with different samples for the antibacterial test with or without the treatment of AC (5 V, 100 mHz, 10 min). The concentrations of MOF@EV were chosen as 0, 1, 2, 4, 8, 16, 32 and 64 µg/mL. The antibacterial test included four groups: (1) PBS (control group), (2) PBS with AC treatment, (3) MOF@EV, and (4) MOF@EV with AC treatment. Finally, all bacterial suspensions were diluted 20 times, and 50 µL of each bacterial solution were plated on LB agar plates at 37 ℃ for 24 h. The colonies were counted and recorded to verify the antibacterial performance *in vitro*.

**1.8 Live/dead bacterial staining analysis**

Live/Dead bacterial staining analysis included six groups: (1) PBS (control group), (2) PBS with AC treatment, (3) MOF, (4) MOF with AC treatment, (5) MOF@EV, (6) MOF@EV with AC treatment. *S. aureus* (1 × 10^8^ CFU/mL) received different treatments and were centrifuged at 3000 rpm for 4 min at 4 ℃. Then, the precipitates were washed and resuspended with 30 μL of PBS at the final density of 1 × 10^9^ CFU/mL. The pre-treated bacteria were then stained with a mixture of SYTO 9 and propidium iodide (PI) for 15 min at room temperature. The SYTO 9/PI-stained bacteria were transferred to the confocal dishes and imaged using a CLSM (Leica, SP8) with a 100-fold objective.

**1.9 *In vitro* antibiofilm properties of MOF@EV**

Agar plate counting method was employed to evaluate the anti-biofilm efficacy of MOF@EV. Biofilms were incubated with MOF@EV at different concentrations of (0, 4, 8, 16, 32, and 64 µg/mL) for 30 min, followed by treatment with or without AC (10 V, 10 min). Then, the pre-treated biofilms were washed and resuspended in PBS, and 50 µL of the bacterial suspensions were cultured on LB agar plates. After diluting 40-fold, the plates were incubated at 37 ℃ for 24 h, and the experimental groups included PBS (as control), MOF, MOF@EV, PBS + AC, MOF + AC, and MOF@EV + AC. The concentration of MOF was maintained at 32 µg/mL. The colonies on the plates were counted and recorded.

**1.10 Live/dead staining of biofilm**

*S. aureus* (1 × 10^8^ CFU/mL, 2 mL) were incubated in LB medium for 2 days at 37 ℃ to form mature biofilms. The biofilms were washed 3 times with PBS and divided into six groups, including PBS (as control), MOF (32 µg/mL), MOF@EV (32 µg/mL), PBS + AC (10 V, 10 min), MOF (32 µg/mL) + AC (10 V, 10 min), and MOF@EV (32 µg/mL) + AC (10 V, 10 min). To evaluate the biofilm viability, PI (20 μL, 30 μM) and SYTO 9 (10 μL, 30 μM) were added to each biofilm and incubated in the dark for 15 min. The biofilm suspensions were then imaged using CLSM (Leica, SP8).

**1.11 ROS Measurement in bacteria**

ROS generation in bacteria was monitored using 2,7-dichlorodihydrouorescein diacetate (DCFH-DA) as the fluorescent probe for ROS detection. *S. aureus* (1 × 10^8^ CFU/mL) suspension was respectively incubated with PBS (as control), MOF (32 µg/mL), and MOF@EV (32 µg/mL) with or without the treatment of AC (10 V, 10 min). Afterward, bacteria were collected by centrifugation and then stained with DCFH-DA (10 µM, 100 µL) for 30 min at 37 ℃. Subsequently, the bacterial solutions were further centrifuged, washed three times, and finally concentrated to 100 µL with PBS. The fluorescence images of DCFH-DA-incubated bacteria were visualized by CLSM (Leica, SP8).

**1.12 Hemolysis assay of MOF@EV**

Red blood cells (RBCs) extracted from mice were isolated from serum. 1 mL of serum was centrifuged at 2000 rpm for 15 min at 4 ℃, and the supernatant was removed. After washing five times with saline via centrifugation at 1500 rpm for 15 min, RBCs were obtained and then resuspended in 1 mL of saline. MOF@EV in saline (0, 32, 64, 128, 256, 512, 1024 μg/mL) were respectively incubated with RBCs (2 %) for 3 h at 37 ℃. Meanwhile, RBCs treated with saline solution and deionized water were regarded as negative and positive controls, respectively. Subsequently, the absorbance of the supernatant at 540 nm was measured by microplate reader after centrifugation at 2000 rpm for 15 min. The hemolysis rate was calculated by the following equation:^[3]^

$$Hemolysis rate \left( \% \right)=\frac{{OD}_{s}-{OD}_{N}}{{OD}_{P}-{OD}_{N}}\times100\%$$

where OD_S_, OD_N_, and OD_P_ are the values of the samples, the negative control, and the positive control, respectively.

**1.13 Neutralization of toxins**

To explore the neutralization of toxins produced by *S. aureus*, RBCs were centrifuged and washed three times with PBS for further use. 100 μL of *S. aureus* (10^5^ CFU/mL) was incubated with EV (1 mg/mL) in shaker incubator for 24 h, and *S. aureus* in LB was used as control group. After 24 h, supernatant was collected by centrifugation, and then RBC was placed in LB for further 3 h incubation at 37 ℃. Then, the supernatant was collected after centrifugation at 2000 rpm for 5 min, and the absorbance at OD 545 was measured by microplate reader. LB was used as negative control, and toxins secreted by *S. aureus* was served as a positive control.

**1.14 Cytotoxicity evaluation *in vitro***

MTT assay was performed using L929 cells to evaluate the toxicity of MOF@EV *in vitro*. L929 cells with 20,000 cells per well were seeded into a 96-well plate, and incubated in an incubator for 24 h. The culture medium was replaced with fresh medium containing serial dilutions of MOF@EV (0, 16, 32, 64, 128, 256, and 512 µg/mL), and the cells were further incubated for 4 h. Then, the cells were washed completely with PBS. After 20 h, MTT solution (0.5 mg/mL, 100 µL) was added into each well and incubated for an additional 4 h. After the removal of the supernatants, 100 µL of dimethyl sulfoxide (DMSO) was added into each well to dissolve the formazan crystals and then shaken for 10 min. The absorbance at 490 nm was measured using a Bio-Rad Model 680 Microplate Reader (UK).

**1.15 *In vivo* fluorescence (FL) imaging of *S. aureus* infections**

FL imaging was used to monitor the biodistribution of MOF@EV using a subcutaneous abscess model. Female BALB/c mice (6 weeks old, weight 16-20 g) were purchased from Beijing Vital River Laboratory Animal Technology Co., LTD (Beijing, China). The subcutaneous abscess model was established by injecting 100 μL of *S. aureus* (1 × 10^8^ CFU/mL) subcutaneously into the thigh muscles. After 24 h, MOF (100 µL, 1 mg/mL) and MOF@EV (100 µL, 1 mg/mL) were intravenously injected via the tail vein, respectively. The FL signals of the infected sites were monitored using an IVIS Lumina imaging system at scheduled time points (0, 2, 4, 8, 16, and 24 h). After 24 h, the mice in each group were sacrificed, and their organs (heart, liver, spleen, lung, kidney) and S. aureus-infected legs were extracted for FL imaging and biodistribution analysis. All animal experiments were approved by the Ethical Committee of the Chinese Academy of Medical Sciences and Peking Union Medical College.

**1.16 Elimination of subcutaneous abscess *in vivo***

Female BALB/c mice (6 weeks old, 16-20 g) were procured from Beijing Vital River Laboratory Animal Technology Co., LTD (Beijing, China). To assess the biofilm elimination potential of MOF@EV *in vivo*, subcutaneous abscess models infected with *S. aureus* were established using BALB/c mice. 100 µL of Luc-labeled *S. aureus* (1 × 10^8^ CFU/mL) was subcutaneously injected into the thigh. After 48 h of *S. aureus* infection, mice (n = 3) were randomly assigned into six groups to evaluate the antibacterial efficacy of MOF@EV-mediated EDT, including (1) group I: PBS (as control), (2) group II: PBS with AC treatment, (3) group III: MOF (1 mg/mL, 100 μL), (4) group IV: MOF (1 mg/mL, 100 μL) with AC treatment, (5) group V: MOF@EV (1 mg/mL, 100 μL), and (6) group VI: MOF@EV (1 mg/mL, 100 μL) with AC treatment. After 8 h, groups II, IV, and VI were subjected to AC field (10 mV, 10 min). In addition, D-luciferin (15 mg/kg, 100 μL) was given by intraperitoneal injection, and after 5 min, the infected tissues were imaged by an IVIS imaging system to determine the bacterial activity. The body weight of mice was measured daily, and the S. aureus-infected legs of different groups were collected on the last day for homogenization and colony counting. The S. aureus-infected legs of each group were plated on LB agar plates and cultured for 24 h at 37 ℃. The standard plate counting assay was conducted to evaluate bacterial growth *in vivo*.

**1.17 Histopathologic examination**

The infected tissues were collected and then sectioned for hematoxylin and eosin (H&E) staining. Additionally, immunofluorescence staining was performed to assess the extent of the inflammatory response caused by *S. aureus* infection. The tissues from all groups were treated with the primary myeloperoxidase (MPO) antibody (Beyotime) and 4',6-diamidino-2-phenylindole (DAPI, Beyotime). The expression levels of TNF-α and IL-6 at the infected tissues were analyzed by enzyme-linked immunosorbent assay (ELISA) quantification kits.

**1.18 Safety evaluation *in vivo***

Biosafety of MOF@EV was investigated using BALB/c mice, and all groups receiving different treatments were sacrificed after 6 days. The surrounding tissues of the abscesses and major organs (heart, liver, spleen, lung, kidney) were carefully harvested for further histomorphological analysis, fixed with 4 % paraformaldehyde solution, embedded in paraffin, and sectioned into 4 µm thickness. H&E staining was carried out, and the sections were visualized under a microscope to evaluate the biosafety. Blood routine tests were used to evaluate the physiological influence after nanoparticles treatment. These indicators included white blood cell count (WBC), lymphocyte count (Ly), monocyte count (MO), red blood cell count (RBC), hemoglobin level (Hb), mean corpuscular volume (MCV), mean corpuscular hemoglobin (MCH), red cell distribution width (RDW), mean platelet volume (MPV), and neutrophil count (NEUT). Furthermore, the alanine aminotransferase (ALT), serum aspartate aminotransferase (AST), creatinine (CREA), and urea (UREA) levels were also measured by assay kit (Nanjing Jiancheng Bioengineering Institute, Nanjing, China).

**1.19 Statistical analysis**

The experiment data were presented as means ± standard deviation, where they were repeated at least three times. Statistical significance (P < 0.05) was evaluated by one-way ANOVA using the Tukey post-test. In all tests, the statistical significance for the tests was set at **P* < 0.05, ***P* < 0.01, and ****P* < 0.001. Statistical analysis was performed using Origin 2019.


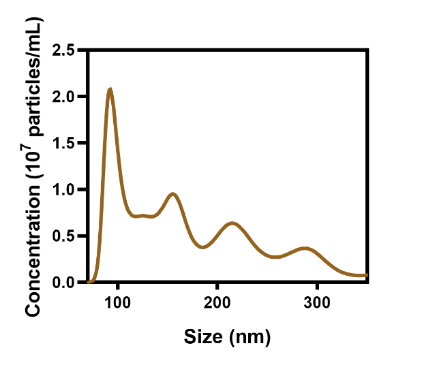


**Figure S1**. Size distribution of EVs measured by Nanoparticle Tracking Analysis (NTA).


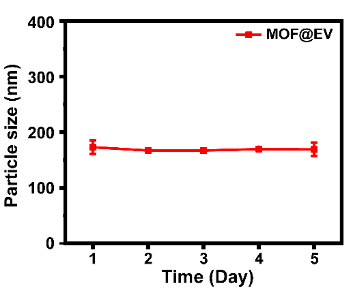


**Figure S2**. The particle size changes of MOF@EV in PBS during 5 days (*n* = 3).


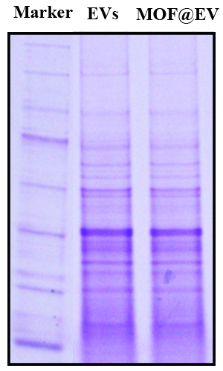


**Figure S3**. Sodium dodecyl sulfate polyacrylamide gel electrophoresis (SDS-PAGE) analysis of EVs and MOF@EV.


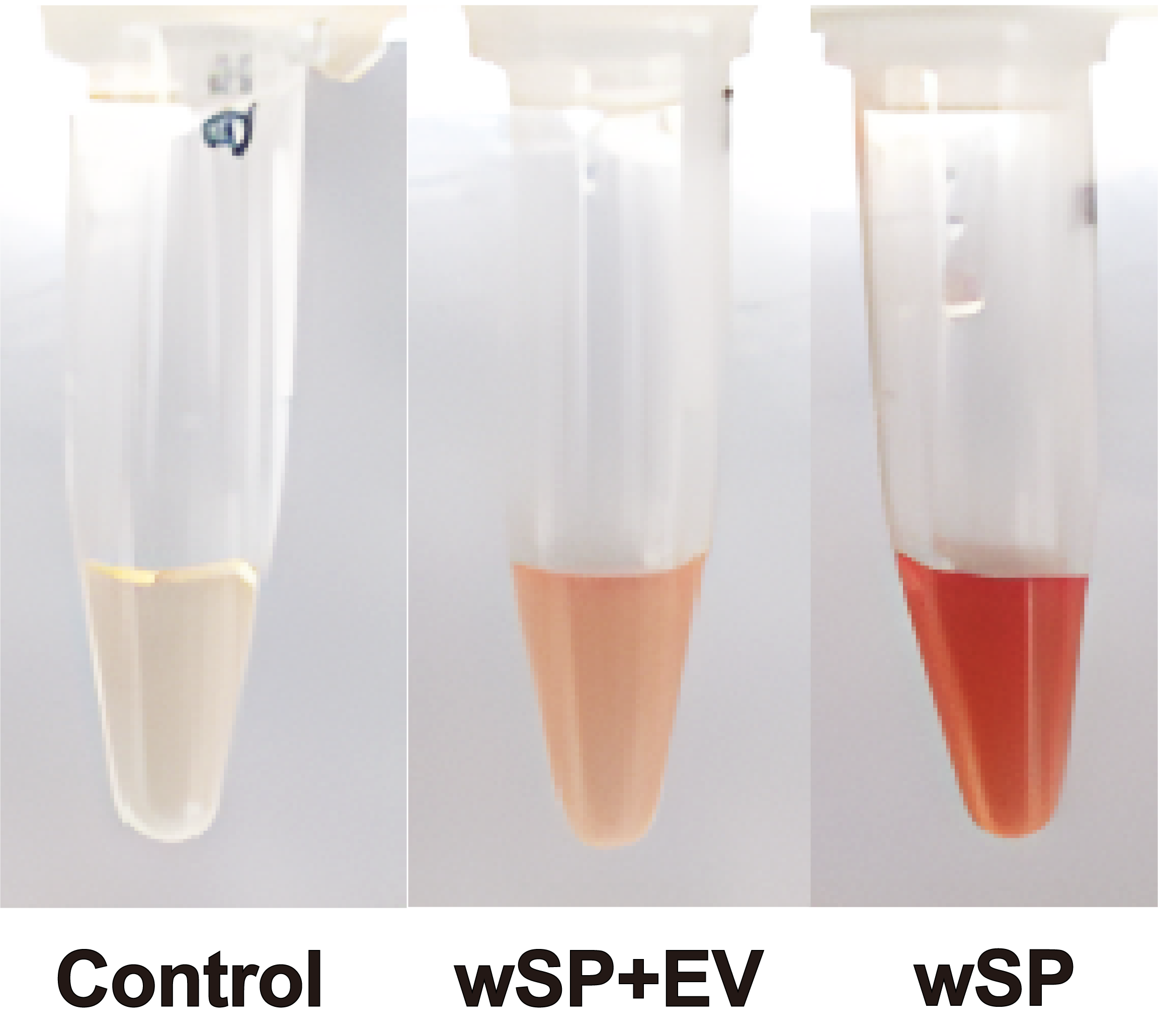


**Figure S4**. Hemolysis photographs of RBCs after different treatments.


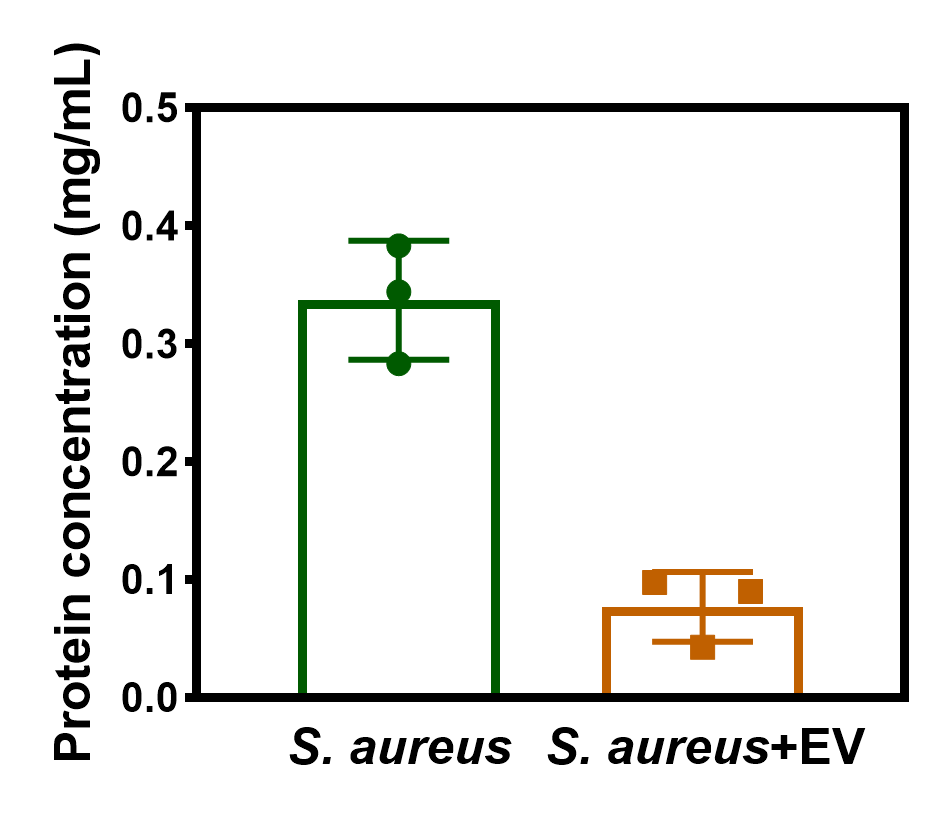


**Figure S5**. The concentration of bacterial toxins measured by BCA protein assay kit after receiving different treatments (*n* = 3).


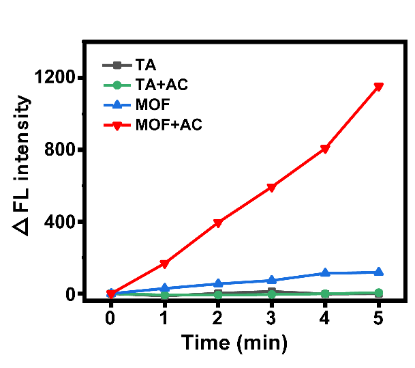


**Figure S6**. Determination of •OH generation using terephthalic acid (TA) as the fluorescent probe.


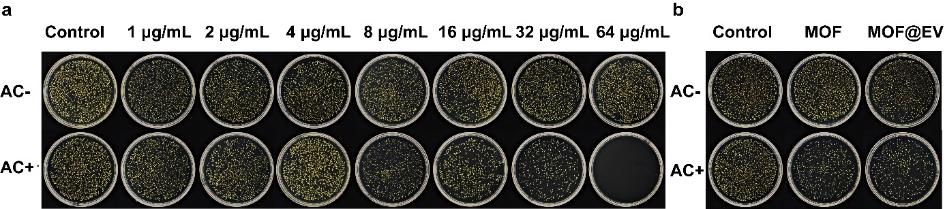


**Figure S7**. *In vitro* antibacterial activity of MOF@EV. (a) The plates of bacteria treated with MOF@EV at different concentrations. (b) The plates of bacteria after different treatments (*n* = 3).





**Figure S8**. Quantitative analysis of TNF-α (a) and IL-6 (b) at subcutaneous abscesses after different treatments (*n* = 3).


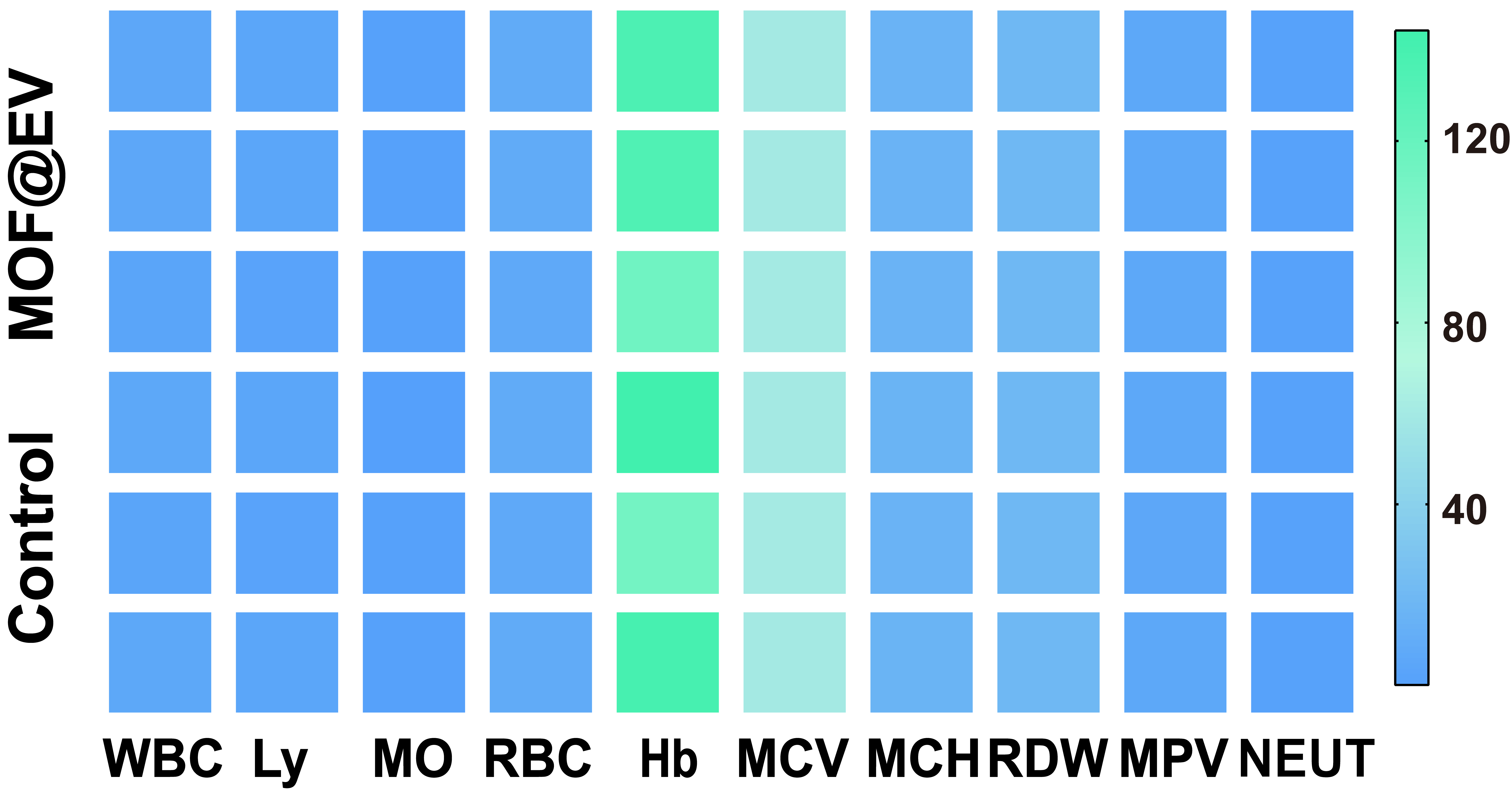


**Figure S9**. Blood routine indexes of different groups designed for heat map (*n* = 3).


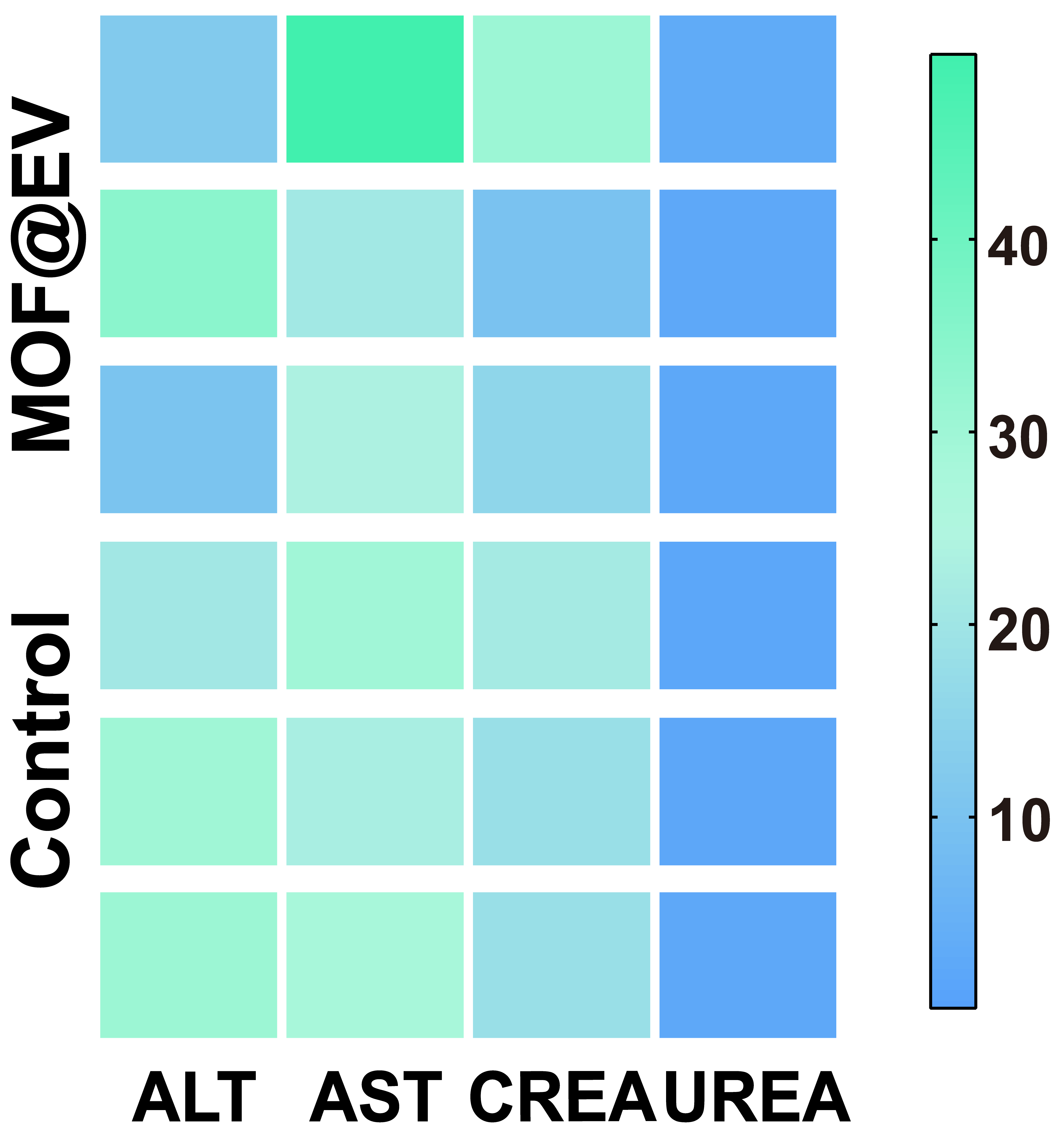


**Figure S10**. Serum levels of ALT, AST, CREA, and UREA in different groups designed for heat map (*n* = 3).


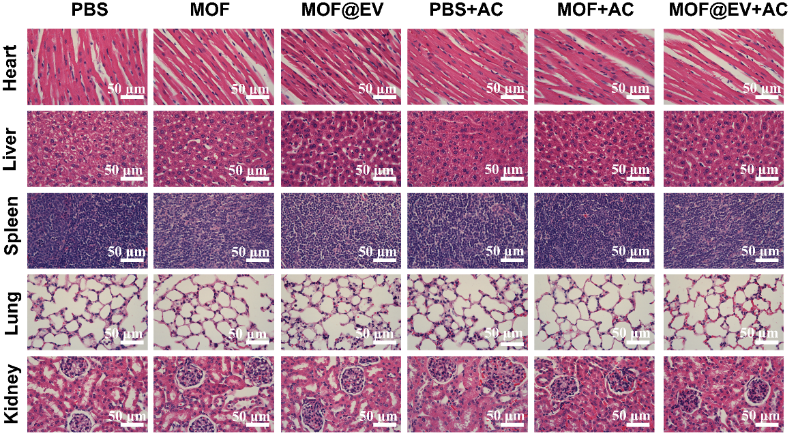


**Figure S11**. H&E staining images of heart, liver, spleen, lung, and kidney of the mice after different treatments.

**References**

[1] J. Park, Q. Jiang, D. Feng, L. Mao, H. Zhou, *J. Am. Chem. Soc*. **2016**, *138*, 3518-3525.

[2] M. Zhang, E. Viennois, M. Prasad, Y. Zhang, L. Wang, Z. Zhang, M. Han, B. Xiao, C. Xu, S. Srinivasan, D. Merlin, *Biomaterials* **2016**, *101*, 321-340.

[3] F. Wang, H. Yuan, J. Shen, Z. Li, J. Li, P. Luo, Q. Zhang, W. Huang, X. Wang, J. Ma, W. Zhang, C. Fu, J. Sun, X. Sun, J. Wang, and W. Xiao, *ACS Mat. Lett.* **2024**, *6*, 1304-1316.
